# Changes in nuclear and cytoplasmic microRNA distribution in response to hypoxic stress

**DOI:** 10.1038/s41598-019-46841-1

**Published:** 2019-07-17

**Authors:** Tiia A. Turunen, Thomas C. Roberts, Pia Laitinen, Mari-Anna Väänänen, Paula Korhonen, Tarja Malm, Seppo Ylä-Herttuala, Mikko P. Turunen

**Affiliations:** 1 A.I. Virtanen Institute for Molecular Sciences, University of Eastern Finland, Yliopistonranta 1E, 70210 Kuopio, Finland; 2 Department of Paediatrics, University of Oxford, South Parks Road, Oxford, OX1 3QX UK; 3 Sanford Burnham Prebys Medical Discovery Institute, Development, Aging and Regeneration Program, 10901 North Torrey Pines Road, La Jolla, CA 92037 USA; 4 Heart Center and Gene Therapy Unit, Kuopio University Hospital, PO Box 100, 70029 KUH Kuopio, Finland

**Keywords:** Non-coding RNAs, Molecular medicine

## Abstract

MicroRNAs (miRNAs) are small non-coding RNAs that have well-characterized roles in cytoplasmic gene regulation, where they act by binding to mRNA transcripts and inhibiting their translation (i.e. post-transcriptional gene silencing, PTGS). However, miRNAs have also been implicated in transcriptional gene regulation and alternative splicing, events that are restricted to the cell nucleus. Here we performed nuclear-cytoplasmic fractionation in a mouse endothelial cell line and characterized the localization of miRNAs in response to hypoxia using small RNA sequencing. A highly diverse population of abundant miRNA species was detected in the nucleus, of which the majority (56%) was found to be preferentially localized in one compartment or the other. Induction of hypoxia resulted in changes in miRNA levels in both nuclear and cytoplasmic compartments, with the majority of changes being restricted to one location and not the other. Notably, the classical hypoxamiR (miR-210-3p) was highly up-regulated in the nuclear compartment after hypoxic stimulus. These findings reveal a previously unappreciated level of molecular complexity in the physiological response occurring in ischemic tissue. Furthermore, widespread differential miRNA expression in the nucleus strongly suggests that these small RNAs are likely to perform extensive nuclear regulatory functions in the general case.

## Introduction

The canonical view of microRNA (miRNA) function is that these small RNA molecules typically repress gene expression by targeting the mRNA 3′ untranslated region (3′ UTR) in order to induce post-transcriptional gene silencing (PTGS)^1^. A growing body of evidence suggests that miRNA functionality may be much more complex than this canonical paradigm, and recent studies have suggested multiple novel modes of action for small RNAs^2–5^. For example, small RNA-mediated transcriptional gene activation (TGA) has been reported in some cases, whereby synthetic small RNAs or endogenous miRNAs targeted to gene promoters can induce increases in gene expression^6,7^. TGA involves an epigenetic mechanism, although a detailed understanding of this phenomenon has thus far remained elusive^8^.

We have previously shown that short hairpin RNA (shRNA)-mediated transcriptional activation of the *Vegfa* promoter is of therapeutic benefit in murine models of hindlimb ischemia^9^ and myocardial infarction^10^. These observations suggested that shRNAs may function by mimicking endogenous miRNAs. To date, intracellular miRNA localization has been largely overlooked, and only a handful of publications have performed global miRNA analysis in nuclear and cytoplasmic fractions. Conversely, the majority of citations focusing on miRNA function have not addressed the issue of subcellular localization. Previous reports have analyzed the subcellular distribution of small RNAs in 5-8F^11^ and HTC116 cells^12^ and showed that the majority of miRNAs, regardless of their sequence, are shuttled to nucleus. Some sequence motifs have been demonstrated to affect miRNA subcellular distribution^13,14^, but other factors may also direct miRNA localization patterns. For example, Khudayberdiev *et al*., observed that nuclear accumulation of miRNAs may be associated with global down-regulation of miRNA levels occurring during neuronal development^15^. Importantly, components of the RNA interference machinery have been observed to be functional in the nucleus, although small RNA loading is restricted to the cytoplasm^16^. While several studies have profiled miRNA expression in the nucleus, no study has to date applied this analysis to cells undergoing physiological transitions (i.e. during cellular differentiation, or in response to environmental stimulus).

Hypoxia is a severe pathophysiological condition observed in multiple diseases (e.g. myocardial infarction and cerebral ischemia) where it impairs tissue function, resulting in a rapid and complex response at the molecular and cellular level. In the present study, we selected hypoxia as a prototypical, medically-relevant, pathophysiological stimulus that is known to lead to changes in gene and miRNA expression^17^. To investigate the extent to which mature miRNAs are present in the nucleus, we performed high throughput sequencing of small RNAs (sRNA-seq) in nuclear and cytoplasmic fractions derived from endothelial cells after the induction of hypoxia. This analysis has identified multiple miRNAs that are enriched in either the nucleus or cytoplasm. Furthermore, we show for the first time, that specific miRNAs are differentially expressed in a subcellular location restricted manner in response to hypoxic stimulus. These findings suggest that miRNAs likely execute nuclear functions in addition to their cytoplasmic PTGS activity, and reveal a previously unappreciated level of complexity in the cellular response to hypoxia.

## Results

### Isolation of nuclear and cytoplasmic fractions from endothelial cells

**Figure 1:**
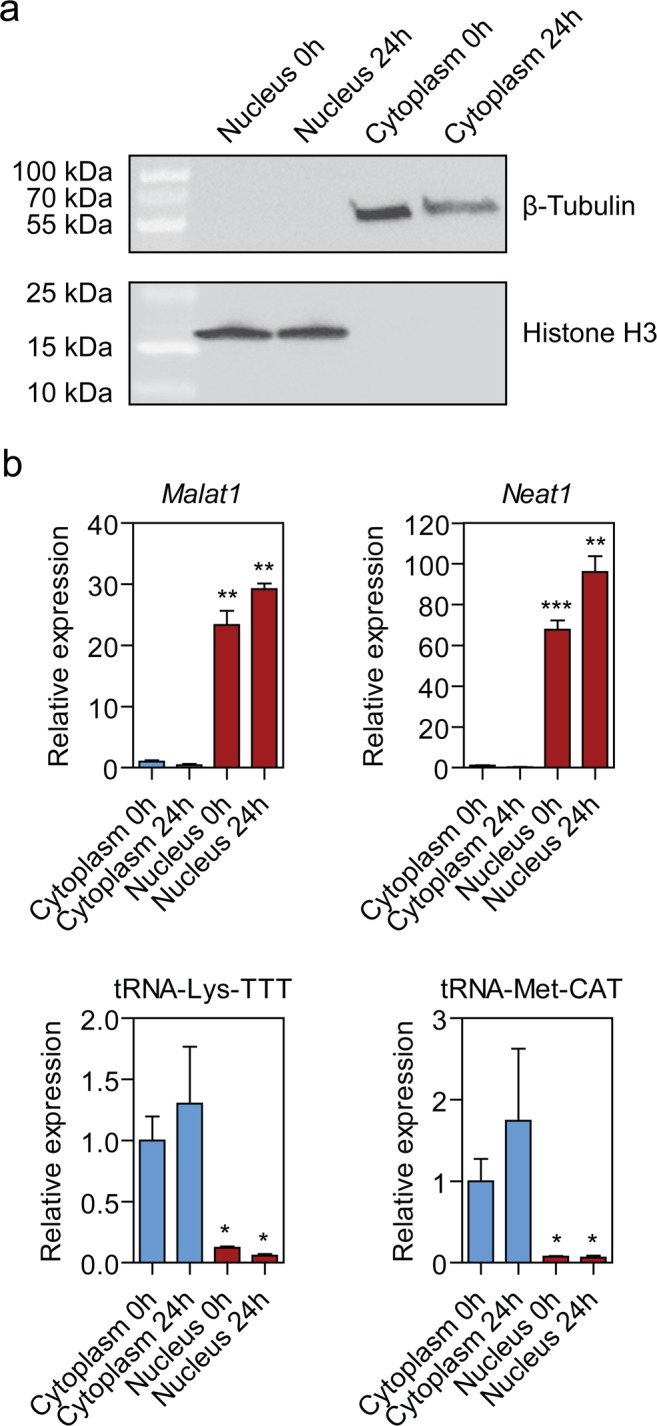
Isolation of nuclear and cytoplasmic fractions. (**a**) Western blot analysis of cytoplasmic (β-tubulin) and nuclear (histone H3) marker protein expression. (**b**) RT-qPCR analysis of nuclear-enriched lncRNAs (*Malat1* and *Neat1*) or cytoplasm-enriched tRNAs (tRNA-Lys-TTT and tRNA-Met-CAT). Values are mean ± SEM, *n* = 3–4, *P < 0.05, **P < 0.01, ***P < 0.001 (*t*-test with Welch correction for unequal variance, comparisons are to the matched group-matched Cytoplasmic fraction).

The murine C166 endothelial cell line was cultured under normal conditions or exposed to hypoxia (1% oxygen) for 24 h. Cells were separated into nuclear and cytoplasmic fractions according to the protocol described by Gagnon *et al*., and RNA and proteins were extracted from each sample^18^ (Supplementary Fig. S1). Fraction purity was confirmed by western blot using antibodies against nuclear (histone H3) and cytoplasmic (β-tubulin) marker proteins (Figs. 1a and S2). Similarly, the nuclear-enriched long non-coding RNAs (lncRNAs) *Malat1* and *Neat1*^19^ were found to be 37- and 125-fold enriched in the nucleus respectively, as determined by RT-qPCR (Fig. 1b). Conversely, transfer RNAs (tRNA) tRNA-Lys-TTT and tRNA-Met-CAT were found to be 12- and 17-fold enriched in the cytoplasm, respectively (Fig. 1b), consistent with successful subcellular fractionation.

### Identification of nuclear and cytoplasmic enriched miRNAs

**Figure 2:**
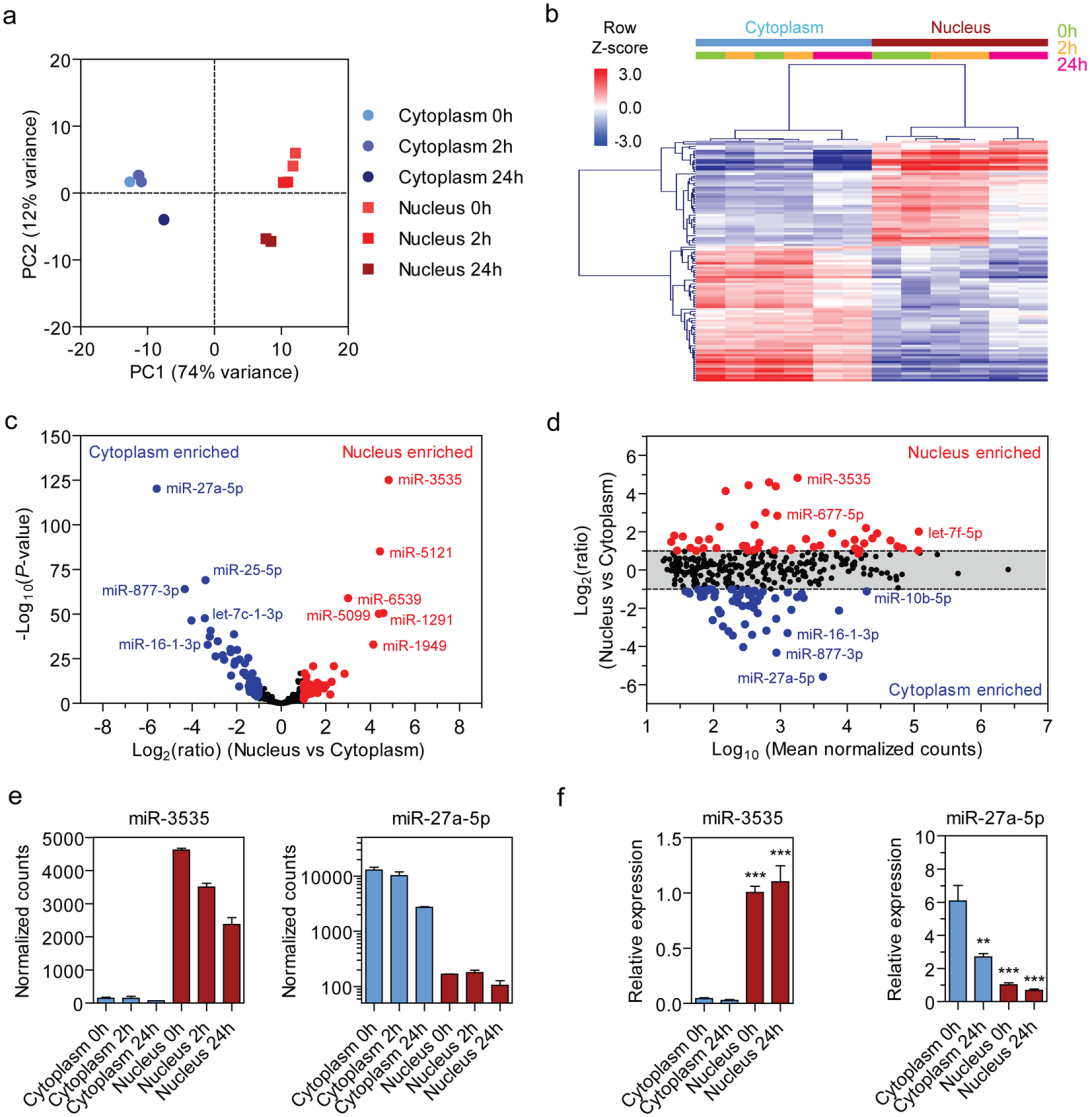
Nuclear and cytoplasmic small RNAs. (**a**) Principal component analysis (PCA) shows tight clustering of biological replicates. Principal component one (PC1) shows the separation between nuclear and cytoplasmic samples (74% of variation), whereas PC2 depicts the response to hypoxic stress (12% of variation). (**b**) Hierarchical clustering of differential expression between nuclear and cytoplasmic samples (adjusted *P* < 0.01, fold change ≥ |2|). Scale bars show mean-centered log_2_ normalized counts (row Z-score) where red and blue indicate higher and lower than mean abundance respectively. (**c**) Volcano plot depicting nuclear and cytoplasmic miRNA enrichment. (**d**) MA plot analysis of location-enriched miRNAs showing that differentially nuclear/cytoplasm-enriched miRNAs were observed over a broad range of absolute expression values. (**e**) The two most location-enriched miRNAs across the dataset were miR-3535 (in the nucleus) and miR-27a-5p (in the cytoplasm). (**f**) miRNA-expression levels of nuclear (miR-3535) and cytoplasmic (miR-27a-5p) miRNAs identified by miRNA-seq were validated by RT-qPCR. miRNA levels were normalized to miR-186–5p and the mean value of the Nucleus 0 h group scaled to a value of one. Values are mean ± SEM, *n* = 2 (sRNA-seq), *n* = 3 (RT-qPCR), *P < 0.05, **P < 0.01, ***P < 0.001 (one-way ANOVA with Bonferroni *post hoc* test, comparisons are to the Cytoplasm 0 h group).

C166 cells were cultured in either normoxic or hypoxic conditions (for 2 h and 24 h) and RNA from nuclear and cytoplasmic fractions analyzed by sRNA-seq (*n* = 2) (Supplementary Fig. S1). 214 million reads were generated in total, and library sizes were found to be comparable between all samples (Supplementary Fig. S3). The data were filtered to remove low abundance miRNAs (<10 reads in each library) after which 350 miRNAs remained. Statistical differences between samples were tested using DESeq2^20^ and libraries were visualized by Principal Component Analysis (PCA) (Fig. 2a) which showed tight clustering of biological replicates. Nuclear and cytoplasmic samples were clearly separated in principal component one (which contained 74% of the variation in the dataset), whereas the response to hypoxic stress was reflected in principal component 2 (12% of the variance).

Nuclear and cytoplasmic enrichment was determined by differential expression analysis of all nuclear libraries against all cytoplasmic libraries and visualized by hierarchical clustering (Fig. 2b) and a volcano plot (Fig. 2c). 196 miRNAs (56% of all miRNAs) were significantly different (*P* < 0.01) between nucleus and cytoplasm, suggesting that the majority of miRNAs are preferentially enriched in one of the compartments. Of these, 105 miRNAs were enriched in either compartment by ≥ 2-fold (i.e. 46 enriched in the nucleus, and 59 enriched in the cytoplasm). MA plot analysis showed that differentially enriched miRNAs were found across a broad range of absolute expression (i.e. counts) values (Fig. 2d). The most strongly location-enriched miRNAs were miR-3535 (27.9-fold enriched in the nucleus) and miR-27a-5p (48-fold enriched in the cytoplasm) (Fig. 2e). Differential nuclear-cytoplasmic distribution of these miRNAs was further confirmed by RT-qPCR, where additional replicate cultures were analyzed to increase statistical power and confirm reproducibility of these findings (Fig. 2f). RT-qPCR data were normalized to the levels of miR-186-5p which was determined to be the most stably expressed miRNA across all conditions using the NormFinder method^21^.

### miRNAs are differentially expressed in distinct subcellular compartments

We next sought to identify miRNAs that were differentially expressed in response to hypoxic stress.

Nuclear and cytoplasmic libraries were considered separately, and differential expression determined between time points. Using a fold-change cut-off of 2, and a stringent statistical cut-off of *P* < 0.01 (Benjamini-Hochberg adjusted), 54 and 35 significantly different miRNAs were called in the nuclear and cytoplasmic libraries respectively for the 24 h vs 0 h comparison. In contrast, no significant differences were called in the 2 h vs 0 h comparison for either nuclear or cytoplasmic fractions.

**Figure 3:**
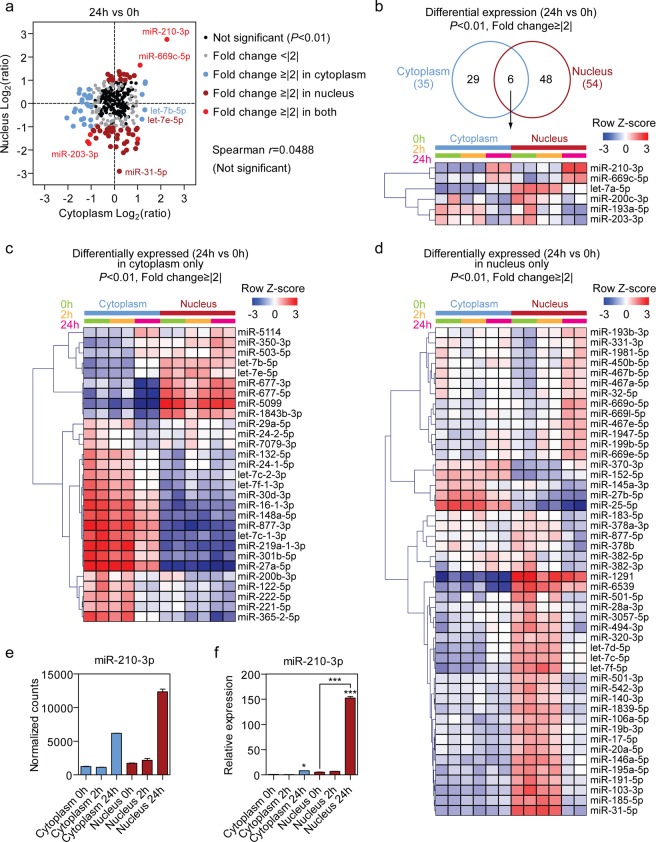
Differential expression of nuclear and cytoplasmic miRNAs upon hypoxic stimulus. (**a**) Comparison of expression ratios for nuclear and cytoplasmic libraries (0 h vs 24 h hypoxia). No significant correlation was observed between the nuclear and cytoplasmic expression ratios. (**b**) Heatmap of common differentially expressed miRNAs upon hypoxia in both nuclear and cytoplasmic samples. (**c**) Heatmap of the differentially expressed miRNAs in cytoplasmic fraction only. (**d**) Heatmap of the differentially expressed miRNAs in nuclear fraction only. Scale bars show mean-centered log_2_ normalized counts (row Z-score) where red and blue indicate higher and lower than mean abundance respectively. (**e**) miR-210-3p was identified to be highly up-regulated upon hypoxia in the nuclear fraction. (**f**) miR-210-3p expression in nuclear and cytoplasmic extracts was validated with RT-qPCR. miRNA-levels were normalized to miR-186-5p levels and the mean value of the Cytoplasm 0 h group scaled to a value of one. Values are mean ± SEM, *n* = 2 (sRNA-seq), *n* = 3 (RT-qPCR), *P < 0.05, **P < 0.01, ***P < 0.001 (one-way ANOVA and Bonferroni *post hoc* test, statistical comparisons are to the Cytoplasm 0 h group unless otherwise indicated).

**Figure 4:**
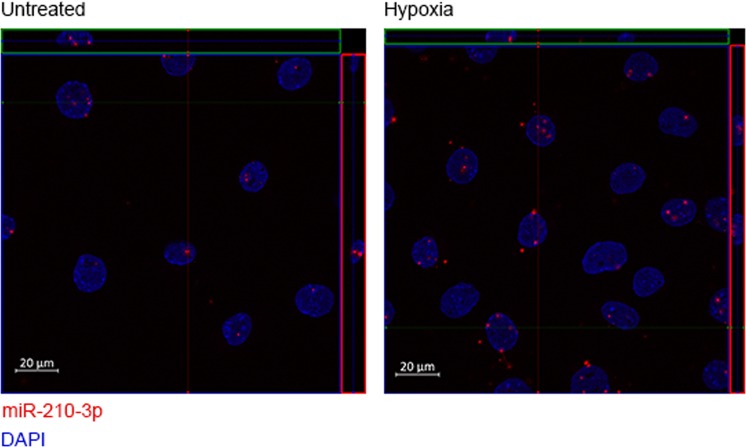
miR-210-3p localization upon hypoxic stimulus. miR-210-3p localization in C166 cells in normoxia and hypoxia (24 h) using fluorescent *in situ* hybridization (FISH). miR-210-3p is found in nucleus in normoxia and hypoxia. DAPI was used to stain nuclei of the cells. miR-210-3p is stained in red. To better display the co-localization of nuclear and miR-210-3p staining, areas within green and red rectangles show the view to the confocal microscopy image stack from the side.

Expression ratios for nuclear and cytoplasmic libraries were not correlated (Pearson *r* = 0.0488, Spearman *r* = 0.0912, *P* > 0.05) (Fig. 3a), indicating that differential miRNA expression typically occurred exclusively in one fraction and not the other. Interestingly, the majority of differentially expressed miRNAs were down-regulated under hypoxic conditions. Only 6 miRNAs were identified as exceptions that were differentially expressed in both nucleus and cytoplasm after 24 h of hypoxic stress (Fig. 3b). These included 2 up-regulated miRNAs (miR-210-3p and miR-669c-5p) and 4 down-regulated (let-7a-5p, miR-200c-3p, miR-193a-5p and miR-203-3p). In the cytoplasm, 29 miRNAs were differentially expressed (Fig. 3c) (5 up-regulated and 24 down-regulated). In the nucleus, 48 miRNAs were differentially expressed (Fig. 3d) (13 up-regulated and 35 down-regulated). Surprisingly, miR-210-3p, a well-described hypoxamiR^22^, was observed to be highly up-regulated in the nuclear fraction in the sRNA-seq data (Fig. 3e). This finding was confirmed by RT-qPCR (Fig. 3f). Fluorescent *in situ* hybridization (FISH) was used to analyze miR-210-3p localization (Fig. 4). Detected signal in confocal microscopy for miR-210-3p was punctate and could be observed in the nuclei of C166 cells both in normoxia and hypoxia (24 h). Qualitative assessment of miR-210-3p puncta also correspond with the increase in miR-210 levels observed by RT-qPCR. miR-210-3p is known to have a role in several pathways related to the low cellular oxygen and has been shown to regulate *EFNA3*, *E2F3*, *HOXA3*, *HIP1*, *BDNF*, *KCMF1* and *NDUFA4P1* by canonical PTGS^23,24^. Furthermore, miR-210-3p was previously shown to interact with is *XIST*, a lncRNA located in the nucleus which is responsible for the initiation of X chromosome inactivation^25^. Together, these data suggest that miR-210-3p likely regulates nuclear processes, in addition to well-established cytoplasmic PTGS mechanisms.

To complement the analysis in C166 cells, we validated the most interesting miRNAs in other mouse cell lines by RT-qPCR. The expression of the miRNAs with nuclear and cytoplasmic localization, miR-3535 and miR-1291 (nuclear) and miR-27a-5p (cytoplasmic), was also observed to be similar in the cell lines MS1 (pancreatic endothelial), MOVAS (smooth muscle) and C2C12 (skeletal muscle) (Supplementary Fig. S4). In addition, miR-210-3p was upregulated in hypoxia in the cytoplasmic and nuclear fractions of these cells in a similar fashion as to that observed in C166 cells. Overall, these results demonstrate that the nuclear-cytoplasmic distribution of certain location-enriched miRNAs, and the nuclear up-regulation of miR-210-3p are similar between different cell lines derived from distinct lineages.

sRNA-seq analysis was performed using equal amounts of input RNA for each sample, and the RT-qPCR analysis performed above normalized according to a stable reference gene identified from the sequencing dataset. These experiments therefore assume that the amount of RNA in the nuclear and cytoplasmic fractions is equal. To address this potential confounding factor, RT-qPCR validation experiments were repeated using new C166 cultures in which a synthetic spike oligonucleotide (the *C*. *elegans* miRNA: cel-miR-39) was added to the nuclear and cytoplasmic fractions at the phenol extraction phase. This technical modification allowed for assumption-free RT-qPCR normalization independent of any difference in total RNA or total miRNA content between the nuclear and cytoplasmic fractions. Results for the miRNAs-of-interest (miR-27a-5p, miR-3535, miR-210-3p and miR-1291) were found to be very similar irrespective of whether the data were normalized to the exogenous spike (cel-miR-39) or the endogenous reference (miR-186-5p) (Supplemental Fig. S5). miRNA expression levels and changes upon hypoxia in cytoplasmic and nuclear compartments were similar with both normalization methods used, indicating that the expressional changes observed are not related to possible different total RNA amount of different samples.

### Analysis of sequence motifs associated with enrichment in nuclear and cytoplasmic fractions

**Figure 5:**
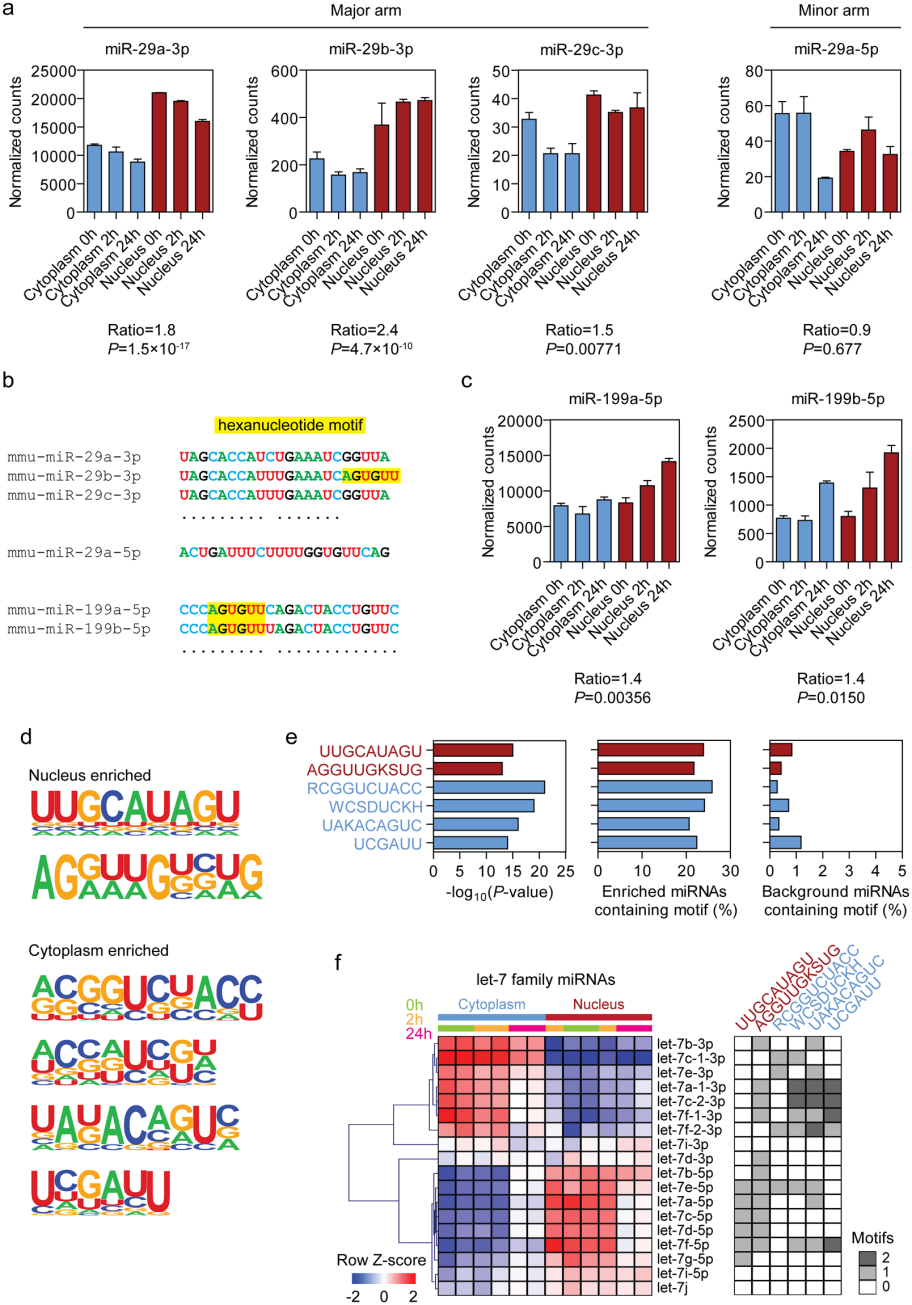
Analysis of putative miRNA sequence motifs affecting subcellular localization. (**a**) sRNA-seq normalized counts values for C166 nuclear and cytoplasmic fractions, with and without hypoxic stress, for the major arms of miR-29 family, and the minor arm of miR-29a-5p. (**b**) Alignments of all detected miR-29 miRNA family members, and miR-199a/b-5p. The positions of the 5ʹ-AGUGUU-3ʹ hexanucleotide motif are indicated in yellow. (**c**) Normalized sRNA-seq counts for miR-199a/b-5p. (**d**) Nuclear and cytoplasmic enrichment sequences identified by *de novo* motif enrichment. (**e**) Output statistics for *de novo* motif discovery; enrichment *P*-values, percentage of enriched miRNAs in containing each motif, and percentage of non-enriched miRNAs (i.e. background) containing each motif. (For ambiguous nucleotides; K = U or G, S = G or C, R = A or G, W = A or U, D = A, G, or U, and H = A, C, or U). (**f**) Heatmap of sRNA-seq data for let-7 family members. Scale bars show mean-centered log_2_ normalized counts (row Z-score) where red and blue indicate higher and lower than mean abundance respectively. Occurrences of each enrichment motif in each family member are indicated in the greyscale heatmap. All values are mean ± SEM, *n* = 2 (sRNA-seq).

Given that it has been previously reported that a hexanucleotide motif (5′-AGUGUU-3′) located at the 3′ terminus of miR-29b-3p was capable for directing this miRNA to the nucleus^14^ we sought to determine the nucleocytoplasmic distribution of miR-29 family members in our sRNA-seq data. miR-29b-3p was found to be significantly enriched in the nucleus by 2.4-fold (Fig. 5a). However, miR-29a-3p and miR-29c-3p (which lack the hexanucleotide motif) were similarly found to be significantly nuclear-enriched, although to a lesser extent; 1.8-fold and 1.5-fold respectively. The minor arm of miR-29a (i.e. miR-29a-5p) was also detected in our sequencing data, and was not significantly enriched in either fraction (Fig. 5a). We next searched for other detected miRNAs containing the hexanucleotide motif. Only two such miRNAs were identified; miR-199a-5p and miR-199b-5p, in which the motif was located towards the 5ʹ end, starting at position 4 (Fig. 5b). These miRNAs were modestly nuclear-enriched by 1.4-fold and 1.5-fold respectively, but dynamically changed their nucleocytoplasmic distribution upon hypoxic stimulus. Furthermore, in the unperturbed normoxic state (time 0 h) miR-199a-5p and miR-199b-5p were expressed at equivalent levels in both compartments (Fig. 5c). Taken together, these findings suggest that the miR-29b-3p hexanucleotide motif is not a *bone fide* nuclear-enrichment signal, at least in this context. Notably, several other studies have failed to detected enrichment of this motif in nuclear sRNA libraries, consistent with the findings reported here^11,12^.

We next used *de novo* motif analysis to identify potential signals that might confer enrichment in either the nucleus or the cytoplasm. Two motifs were significantly enriched in nuclear-localized miRNAs, whereas four motifs were enriched in cytoplasm-localized miRNAs (Fig. 5d,e). Upon inspection of motif annotation results, it became apparent that in many cases, motifs were commonly identified in related miRNA family members. This effect was most strongly apparent for the let-7 family (Fig. 5f). As such, the empirically-determined nuclear enrichment signals 5ʹ-UUGCAUAGU-3ʹ and 5ʹ-AGGUUGKSUG-3ʹ (these motifs also have the potential to overlap) where found to be present in almost all of the let-7 family members that exhibited nuclear enrichment. Interestingly, nuclear enrichment was observed only for the 5ʹ arm (i.e. the dominant arm) of the let-7 family members. In contrast, 3ʹ arm let-7 family members (i.e. the minor arm that is expressed with ~10 times fewer read counts than the dominant arm) were almost exclusively enriched in the cytoplasm, and contained many of the empirically-identified cytoplasmic enrichment motifs. These findings demonstrate that the 5ʹ and 3ʹ arms of let-7 family miRNAs exhibit opposite nucleocytoplasmic localization. The detection of multiple related miRNAs is therefore likely to have skewed the motif enrichment analysis. It is consequently non-trivial to determine if the identified nuclear enrichment motifs are directing their associated miRNAs to distinct subcellular locations, or if they are identified as a consequence of multiple co-regulated miRNAs with high sequence similarity biasing the motif enrichment analysis. Specific details of the *de novo* motif enrichment analysis are described in Supplementary File S1.

## Discussion

In this study we have profiled the subcellular localization of miRNAs in endothelial cells, and identified a set of miRNAs which exhibit nuclear enrichment. Furthermore, induction of hypoxia resulted in differential miRNA expression changes that were spatially restricted to either the nucleus or cytoplasm, or a small number of changes that occurred simultaneously in both compartments. This is the first study to profile subcellular compartment-specific differential miRNA expression, and provides a strong rationale for the investigation of nuclear and cytoplasmic miRNA levels in other biological contexts.

Hypoxia-associated changes in miRNA levels in the nucleus are highly suggestive of functional significance. However, the mechanisms-of-action for nuclear miRNAs are at present incompletely understood. Studies of TGS and TGA mediated by exogenous small RNAs provided early clues that endogenous miRNAs might regulate transcription in a similar manner. Indeed, there have now been multiple studies that have demonstrated miRNA-mediated transcriptional regulation^7,26–33^. It is generally thought that in these instances, the miRNA directs an Argonaute-containing protein complex to non-coding, promoter-associated transcripts^26,27,29^. Exogenous small RNAs have also been shown to regulate pre-mRNA splicing decisions^34–36^, although such effects have yet to be demonstrated for endogenous miRNAs. The observation that siRNAs are capable of silencing nuclear-localized target transcripts implied a similar activity for miRNAs^16,37,38^. Indeed, a recent study by Sarshad *et al*., showed that AGO2 protein is highly expressed in the nuclei of stem cells, where it mediates the silencing of nuclear transcripts, such as pre-mRNAs^39^. Aside from the regulation of gene expression, the miRNA processing machinery has also been implicated in the cellular response to DNA damage^40,41^, the regulation of 3-dimensional chromatin structure^42^, and regulation of the production of intron-included, truncated proteins as a consequence of direct interactions between the miRNA and genomic DNA^43^.

Relative to the enormous research effort directed towards canonical miRNA activity, the nuclear targets and functions of specific miRNAs have so far been largely neglected. Further studies are needed to elucidate the mechanisms through which nuclear miRNAs execute their functions in different cell types and physiological conditions. Given the diversity of functional possibilities this presents a significant task. Analogous to the manner by which the post-transcriptional regulation of mRNA expression and translation can be fine-tuned by multiple miRNAs binding on multiple co-acting sites^44^, multiple non-canonical mechanisms mediated by multiple miRNA may be acting on a gene in the nucleus at any one time. (For example, miRNAs also have the potential to target mRNA coding regions^45^ and intronic sequences in newly transcribed pre-mRNA^39^ simultaneously). One approach is to predict miRNA target sequences in regions that are considered non-canonical targets^46^. However, this strategy does not take into account the transcriptional landscape of the cell of interest, which is expected to be highly lineage specific (e.g. a gene expressed at very high levels might not be susceptible to gene activation). Predictions are also expected to have a high false positive rate, and so extensive experimental validation is required to identify *bona fide* miRNA-target interactions. Alternatively, crosslinking immunoprecipitation techniques can be used to empirically determine miRNA binding events^47,48^. These techniques suffer from the limitation of having relatively low resolution, and of being technically challenging.

miRNAs constitute a promising class of therapeutic target molecules that can be inhibited using antisense oligonucleotides or mimicked using synthetic siRNAs, shRNAs or artificial miRNAs^49^. Our group has previously demonstrated transcriptional regulation of *Vegfa* expression by promoter-targeted shRNAs *in vitro* and *in vivo*^9,10^. Depending on their target site, these shRNAs are able to either up-regulate (TGA) or down-regulate (TGS) *Vegfa* expression^9^. These shRNAs were shown to exert positive therapeutic effects in both hindlimb ischemia and myocardial infarction models^9,10^. Similarly, an siRNA which activates CEBPA expression^50^ is being explored as a treatment for hepatocellular carcinoma with patients currently being enrolled for a phase I clinical trial sponsored by MiNA Therapeutics^51^. As such, nuclear miRNA functions offer new possibilities for the manipulation of gene expression, or may be novel targets in their own right.

Given that altered miRNA expression in hypoxia gives rise to multiple gene expression changes associated with canonical miRNA function^17^, our data suggest that nuclear miRNAs may similarly regulate a wide variety of hypoxia-associated genes through non-canonical mechanisms. This is exemplified by the well-established hypoxamiR miR-210-3p, which was significantly increased in the nucleus of the cells upon hypoxic stimulus, and therefore likely regulates a plethora of nuclear targets. This notion is supported by a previous report showing the interaction between miR-210-3p and the nuclear lncRNA *XIST*^25^. Indeed, interaction between small RNAs and lncRNAs is a common theme. For example, miR-9 has been shown to downregulate *MALAT1* in the nucleus^52^, and promoter-associated transcripts have been shown to regulate transcription of the *VEGFA* locus by acting as targets for TGA-inducing small RNA^53^.

Notably, hypoxic conditions can vary in different physiological contexts with corresponding differences in the cellular gene expression response. This was demonstrated in a recent study in the context of the tumor microenvironment where the commonly-used, continuous, long-term hypoxia condition was compared to a shorter cyclic hypoxia protocol^54^. Previously-described hypoxamiRs were identified in the long-term hypoxia model, but cyclic hypoxic conditions resulted in a different expression pattern, with only 31 common miRNAs between these groups (including miR-210-3p). These observations illustrate the complexity of ncRNA regulation in hypoxia, an issue compounded when considering the possible functions of miRNAs in the nucleus.

Components of the RNAi machinery such as AGO2, Dicer, and GW182 (TRNC6A) have been shown to be present in the nucleus^16^. Given that Dicer processing and RISC loading are generally considered to be restricted to the cytoplasm^16^, this would necessarily require mechanisms for the re-import of miRNAs to the nucleus. The karyopherins exportin-1 (XPO1)^55^ and importin-8 (IPO8)^56^ have been implicated in the transport of miRNA shuttling across the nuclear envelope. Recently, it was also shown the stress-induced response complex (SIRC) (consisting of AGO1, AGO2, YB1, CTCF, FUS, SMAD1, SMAD3, and SMAD4 proteins) is involved in nucleocytoplasmic trafficking of miRNAs in a cellular stress-dependent manner^57^. As such, RNA binding proteins and complexes like SIRC may regulate the subcellular distribution of miRNAs, as we observe also in response to hypoxic stimulus. Some studies have suggested that short sequence motifs might act as nuclear localization signals, as in the case of the 3ʹ terminal hexanucleotide motif in miR-29b-3p, presumably by recognizing specific RNA binding proteins such as ANT2 (ADP/ATP translocase 2)^58^. However, while we did observe a modest nuclear enrichment of miR-29b-3p, the related miRNAs miR-29a-3p and miR-29c-3p which lack the hexanucleotide motif were similarly nuclear-enriched. We have previously suggested an alternative hypothesis whereby miRNAs are non-specifically shuttled between nucleus and cytoplasm and then become enriched in the compartment where their target transcripts are most concentrated^2,59,60^.

In conclusion, this study demonstrates that miRNAs are differentially expressed in the nucleus and cytoplasm in response to hypoxic stress. These findings offer new insights into the molecular response to hypoxia, and for the function of miRNAs in general. Studies of ncRNA networks in gene regulation by our group and others significantly expand our understanding of the role of miRNAs beyond their canonical PTGS functions, and suggest that a vibrant world of ncRNA biology and regulatory potential resides within the nucleus.

## Materials and Methods

### Cell culture

C166 (yolk-sac-derived mouse endothelial cells, ATCC:CRL-2581), C2C12 (skeletal myoblast-derived mouse muscle cells, ATCC:CRL-1772), MS1 (pancreas/islet of Langerhans-derived mouse endothelial cells, ATCC:CRL-2279) or MOVAS (aorta-derived smooth muscle cells, ATCC:CRL-2797) cells were maintained under normal conditions (37 °C, 5% CO_2_). Cells were cultured in Dulbecco’s Modified Eagle’s Medium (DMEM) (Sigma-Aldrich) containing 10% fetal bovine serum (FBS) and 1% penicillin-streptomycin (PS). For hypoxia experiments, cells were cultured in a hypoxia chamber with 1% O_2_, 5% CO_2_ (Baker Ruskinn).

### Western blot

Protein samples were extracted from nuclear and cytoplasmic fractions according to protocol by Gagnon *et al*.,^18^ and with TRI reagent (Molecular Research Center) according to the manufacturer’s instructions. For western blot, equal volumes of extracted protein were loaded on precast gels (Mini-Protean TGX Stain-free Precast Gel, 4–20%, Bio-Rad) and transferred to nitrocellulose membranes (Trans-Blot Turbo Bio-Rad Midi, 0.2 μm nitrocellulose, Bio-Rad). The membranes were blocked with 5% milk for 1.5 hours at room temperature, washed with TBST (0.15 M sodium chloride, 0.050 m TRIS-HCl buffer; 0.05% Tween 20; pH 7.6) and incubated with antibodies against a known nuclear protein (anti-trimethyl-histone H3 (Lys27), Millipore, 1:2,500) and a cytoplasmic protein (anti-β-tubulin, Sigma Aldrich, 1:1,000) overnight at 4 °C. The membranes were washed with TBST and incubated with secondary antibodies (goat anti-rabbit IgG (H + L), HRP-conjugated, Invitrogen; anti-mouse IgG, HRP-conjugated, R&D systems; both 1:5,000) for 1 hour at room temperature. Membranes were analyzed using ECL Plus Western Blotting Substrate (Pierce) and imaged with the ChemiDoc Imaging System (Bio-Rad).

### RNA extraction and small RNA-sequencing

For sRNA-sequencing samples, C166 cells were cultured in hypoxic conditions for 0, 2 or 24 hours and cells were separated into nuclear and cytoplasmic fractions, as described previously^18^. Briefly, the cells were collected from 15 cm plates by scraping and washed once with cold PBS. Cells were pelleted by centrifugation at 700 *g* for 5 minutes and lysed with hypotonic lysis buffer (10 mM Tris (pH 7.5), 10 mM NaCl, 3 mM MgCl2, 0.3% (vol/vol) NP-40, 10% (vol/vol) glycerol) to collect the cytoplasmic fraction. Cytoplasmic RNA was obtained by ethanol precipitation overnight at −20 °C followed by re-extraction using TRI reagent. The remaining nuclear pellet was washed three times with the hypotonic lysis buffer, followed by extraction with TRI reagent according to the manufacturer’s instructions. Libraries were prepared using the NEBNext Multiplex Small RNA Library Prep Set for Illumina kit (New England Biolabs). Equimolar quantities of each library were pooled and small RNA sequencing performed as a service by Exiqon A/S (Vedbaek, Denmark). FASTQ files were trimmed using Trimmomatic^61^ and aligned to reference genome (miRBase release 20) using bowtie2 (v2.1.0)^62^. Differential expression was tested using DESeq2 ^20^ which utilizes the Wald test for determining statistical significance. (Benjamini-Hochberg adjusted *P*-values are reported). Counts data for the full dataset are provided in Supplementary Data S1. sRNA-seq data were analyzed using the NormFinder method^21^ which identified miR-186-5p as being stably expressed between all experimental groups. This miRNAs was subsequently selected as a reference gene for RT-qPCR data normalization. Alternatively, 15 fmol of cel-miR-39 ssRNA was added to the samples during phenol extraction phase and was used as spike-in to calculate relative expression of miRNAs.

### RT-qPCR

Total RNA samples were treated with DNase I, RNase-free (Thermo Fisher Scientific) in order to eliminate genomic DNA contamination. For miRNA analysis, cDNA synthesis was performed using the TaqMan MicroRNA Reverse Transcription Kit (Thermo Fisher Scientific) according to manufacturer’s protocol and analyzed using miRNA-specific TaqMan assays (mmu-miR-27a-5p ID: 002445; mmu-miR-3535 ID: CTEPR23; mmu-miR-186-5p, ID: 002285, mmu-miR-210-3p ID: 000512; mmu-miR-1291, ID: 466942_mat; cel-miR-39-3p, ID: 000200; Thermo Fisher Scientific).

For expression analysis of lncRNAs and tRNAs, cDNA was synthesized using RevertAid Reverse Transcriptase (Thermo Scientific) and random hexamer primers (lncRNA) or gene-specific primers (tRNA) (reverse primers, Supplemental Table 1) and quantified using Maxima SYBR Green/ROX qPCR Master Mix (2×) (Thermo Fisher Scientific). Thermal cycling was performed using a LightCycler480 (Roche) with the following program: 10 min at 95 °C, followed by 50 cycles of 15 s at 95 °C and 60 s at 60 °C. Primers for lncRNA and tRNA analysis are shown in Supplemental Table 1. RT-qPCR data were analyzed using the ΔΔCq method where normalization was available, or the ΔCq method for un-normalized data (validation of nuclear-cytoplasmic fractionation only). Statistical significance was assessed by one-way ANOVA with Bonferroni *post hoc* correction or *t*-test with Welch correction as appropriate (GraphPad Prism 5 (GraphPad Software, La Jolla, CA, USA)).

### miRNA fluorescent *in situ* hybridization (FISH)

For miRNA FISH, C166 cells were seeded on 8-well chamber slides and grown in normoxia or hypoxia (24 h). ViewRNA miRNA ISH Cell Assay Kit (Thermo Fisher Scientific) was used for the hybridizations according to the manufacturer’s protocol. ViewRNA Cell Plus Probe Set (Thermo Fisher Scientific) was used to detect miR-210-3p (assay ID: VM1-10263-VCP, detection label Alexa Fluor 546). Cell nuclei were visualized using DAPI stain. Pictures were taken using ZEISS LSM700 confocal microscope using 40× oil objective and analyzed with ZEN lite blue 2.6 software (ZEISS).

### *de novo* motif enrichment

Sequence motifs were identified in lists of miRNA sequences (enriched in either nucleus or cytoplasm) using HOMER (v4.9.1)^63^. Motifs were restricted to between 6 and 10 nucleotides, and analysis run in strand-specific mode using the findMotifsGenome.pl function. miRNA sequences were subsequently annotated to identify those which contained one or more HOMER-identified motifs using the annotatePeaks.pl function.

## Supplementary information

Supplementary information

Dataset 1

## Data Availability

The data discussed in this publication have been deposited in NCBI’s Gene Expression Omnibus^64^ and are accessible through GEO Series accession number GSE125390 (https://www.ncbi.nlm.nih.gov/geo/query/acc.cgi?acc=GSE125390).
