# Supplementary material for: Changes in nuclear and cytoplasmic microRNA distribution in response to hypoxic stress
Source: Sci Rep. 2019 Jul 17;9:10332. doi: 10.1038/s41598-019-46841-1 (PMC6637125; doi:10.1038/s41598-019-46841-1)
Supplement: Supplementary file 1 — Supplementary information [file 41598_2019_46841_MOESM1_ESM.docx]

**Changes in nuclear and cytoplasmic microRNA distribution in response to hypoxic stress**

Tiia A. Turunen, Thomas C. Roberts^§^, Pia Laitinen^§^, Mari-Anna Väänänen, Paula Korhonen, Tarja Malm, Seppo Ylä-Herttuala and Mikko P. Turunen^*^

**SUPPLEMENTARY INFORMATION**

**Supplementary Data S1**. Full dataset including the counts data and motif analysis.


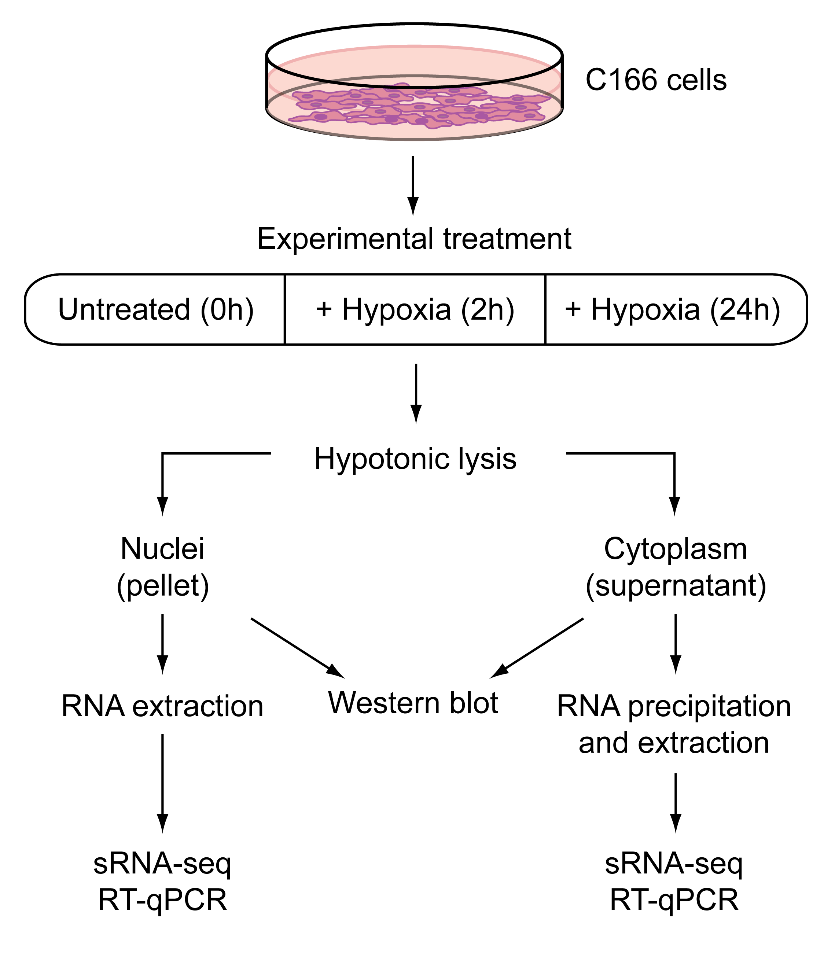


**Supplementary Figure S1.** Experimental design. Schematic of sample preparation. C166 cells were cultured in normoxic conditions or hypoxic conditions for 2 or 24 hours. Nuclear and cytoplasmic fractions were isolated and purity was assessed by Western blot or RT-qPCR. RNA extracts were analyzed by sRNA-seq.


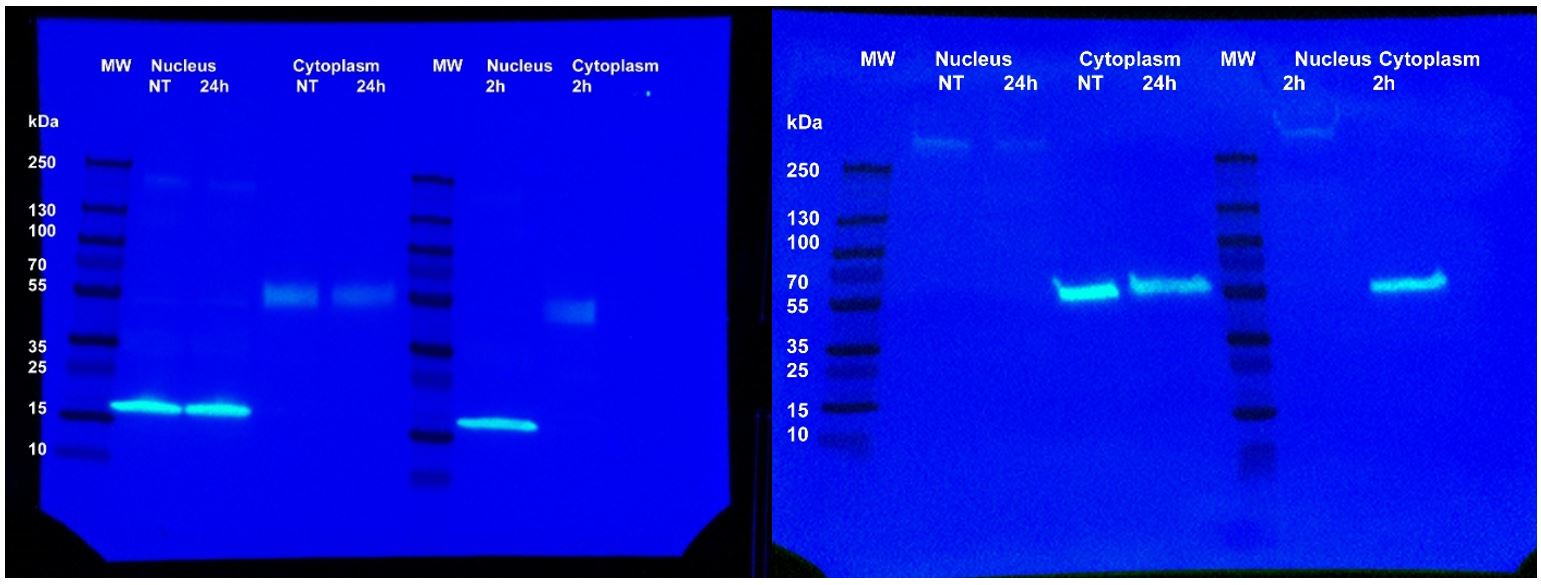


**Supplementary Figure S2.** Whole western blot image for histone H3 (left) and β-tubulin (right).


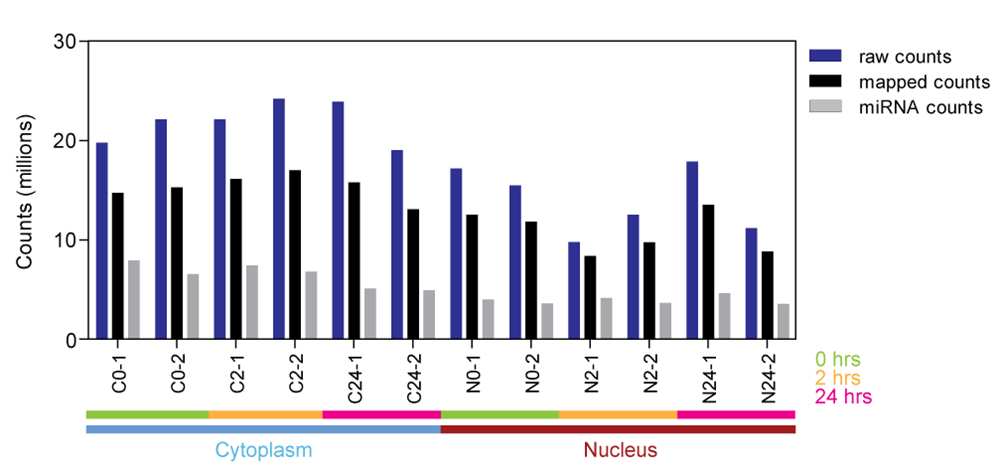


**Supplementary Figure S3.** Sequencing statistics**.** Library sizes, number of mapped reads, and number of miRNA-mapping counts for all samples analyzed in this study.


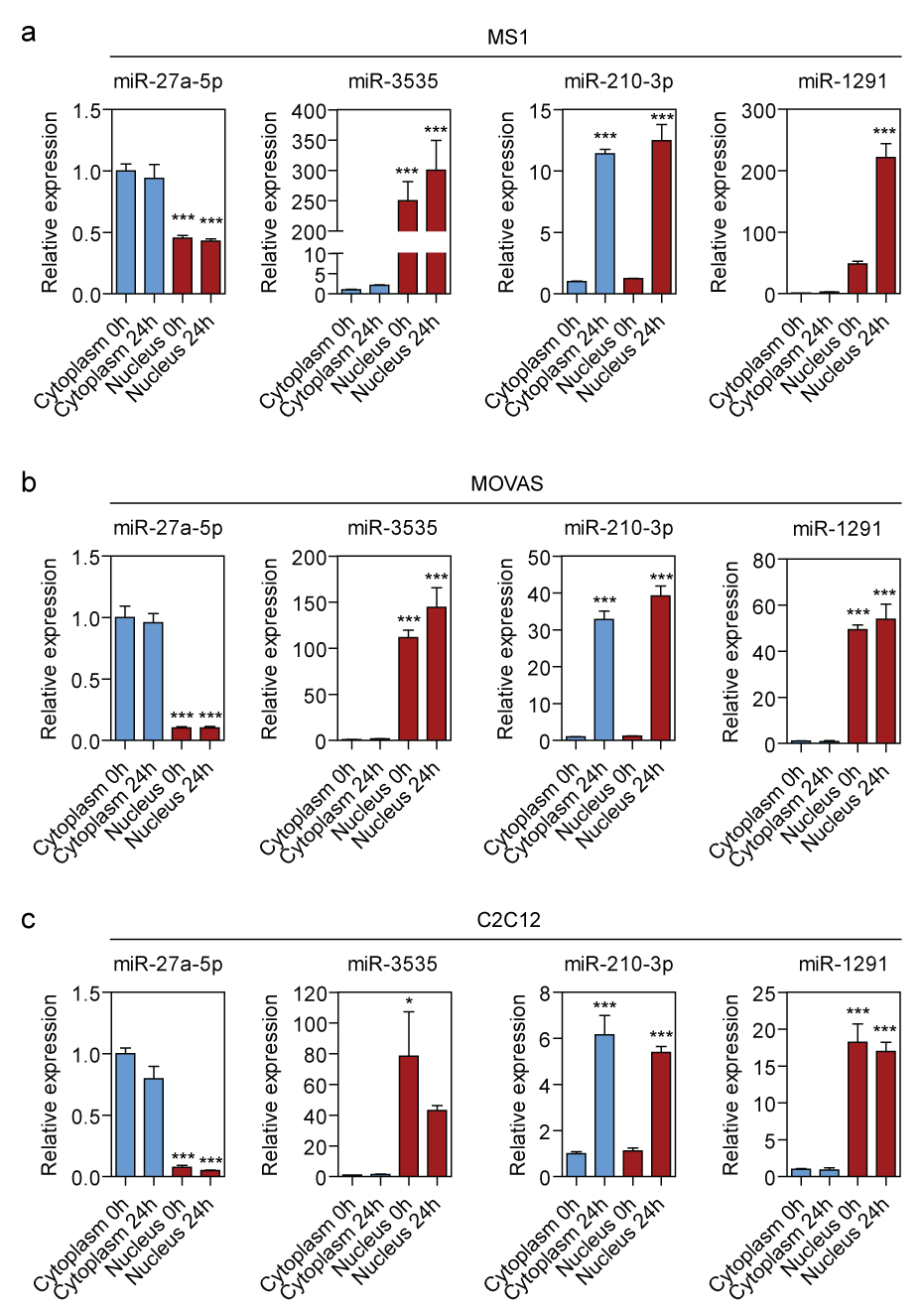


**Supplementary Figure S4.** To validate the results observed in the C166 cells, the expression of the miRNAs that are most expressed in cytoplasm (miR-27a-5p) and in the nucleus (miR-3535, miR-1291) and the most differentially expressed miRNA upon hypoxia in the nucleus (miR-210-3p) in C166 data was validated in three other mouse cell lines MS1 (a), MOVAS (b) and C2C12 (c). miRNA-levels were normalized to miR-186-5p levels and the mean value of the Cytoplasm 0h group scaled to a value of one. Values are mean±SEM, *n*=4, **P*<0.05, ***P*<0.01, ****P*<0.001 (one-way ANOVA and Bonferroni *post hoc* test, statistical comparisons are to the Cytoplasm 0h group).


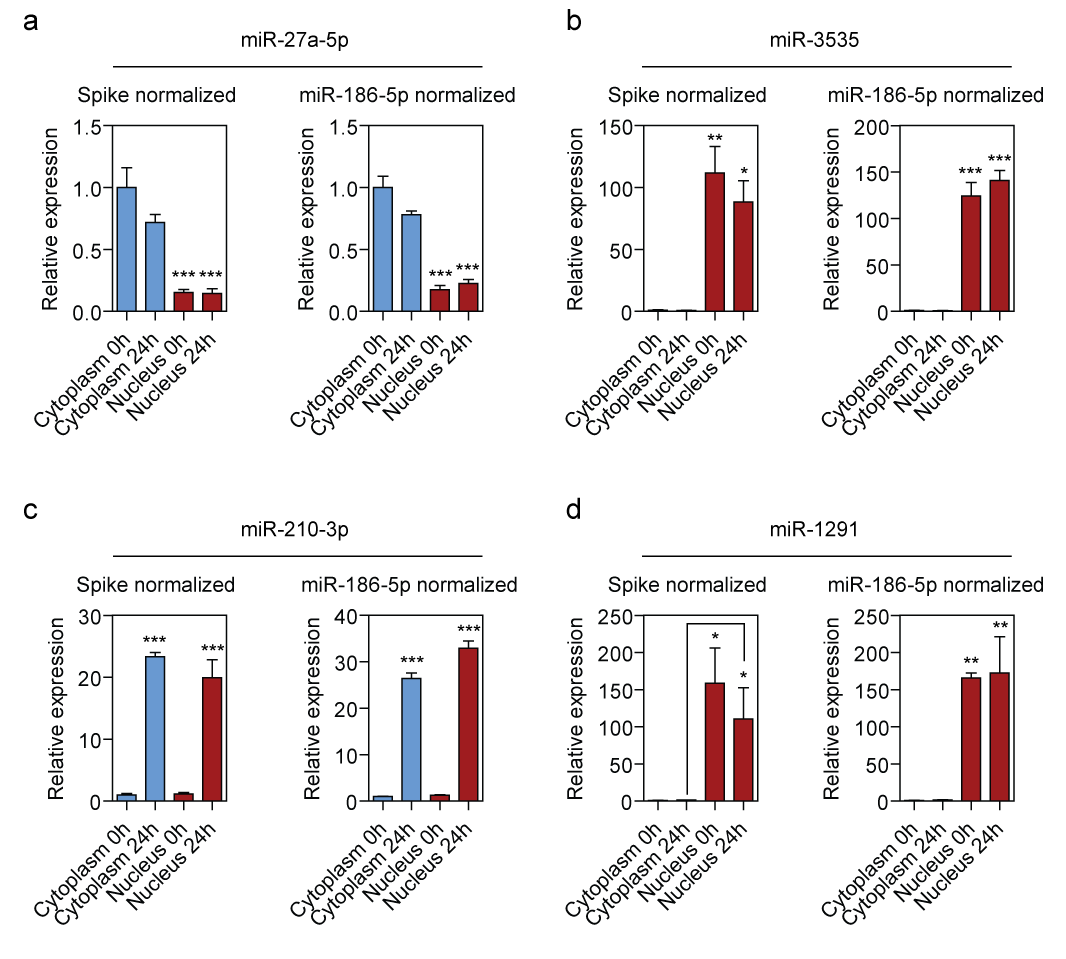


**Supplementary Figure S5.** Evaluation of RT-qPCR normalization methods. C166 samples were spiked with 15 fmol cel-miR-39 RNA in RNA extraction and RT-qPCR performed for miRNAs of interest (a) miR-27a-5p, (b) miR-3535, (c) miR-210-3p and (d) miR-1291 and normalized to either miR-186-5p levels or to the spike (cel-miR-39). Mean value of the Cytoplasm 0h group was scaled to a value of one. Values are mean±SEM, *n*=3, **P*<0.05, ***P*<0.01, ****P*<0.001 (one-way ANOVA and Bonferroni *post hoc* test, statistical comparisons are to the Cytoplasm 0h group, unless otherwise indicated).

**Supplementary Table S1.** PCR primer sequences for lncRNA and tRNA expression analysis. All sequences are 5ʹ to 3ʹ.

|  | **Forward primer sequence** | **Reverse primer sequence** |
| --- | --- | --- |
| ***Malat1*** | GCTGGTAACCGCTGCTATAA | CAGAGAATCCAGACCCAGTAAG |
| ***Neat1*** | CGCTACTGACCACAGACTTTAC | GTTGGATTGGGTCTCCTTCTAC |
| **tRNA-Lys-TTT-1-3** | GCCCGGATAGCTCAGTCG | CGCCCGAACAGGGACTTG |
| **tRNA-Met-CAT-1-2** | GCCTCGTTAGCGCAGTAG | TGCCCCGTGTGAGGATCG |
